# The Adaptation of Cancer Cells to Serum Deprivation Is Mediated by mTOR-Dependent Cholesterol Synthesis

**DOI:** 10.3390/ijms262210932

**Published:** 2025-11-12

**Authors:** Bayansulu Ilyassova, Nargiz Rakhimgerey, Saule Rakhimova, Nazerke Satvaldina, Asset Daniyarov, Ainur Akilzhanova, Ulykbek Kairov, Dinara Begimbetova, Dos D. Sarbassov

**Affiliations:** 1 Department of Biology, School of Sciences and Humanities, Nazarbayev University, Astana 010000, Kazakhstan; bayansulu.ilyassova@nu.edu.kz (B.I.); nargiz.rakhimgerey@nu.edu.kz (N.R.); 2 Center for Life Sciences, National Laboratory Astana, Nazarbayev University, Astana 010000, Kazakhstan; saule.rakhimova@nu.edu.kz (S.R.); nazerke.satvaldina@nu.edu.kz (N.S.); asset.daniyarov@nu.edu.kz (A.D.); akilzhanova@nu.edu.kz (A.A.); ulykbek.kairov@nu.edu.kz (U.K.)

**Keywords:** serum deprivation, protein synthesis, polysomes, cholesterol synthesis, reactive oxygen species, apoptosis, mechanistic target of rapamycin complex 1 (mTORC1)

## Abstract

Cancer cells can sustain survival independently of exogenous growth factors. To investigate their adaptation to serum deprivation, we analyzed transcriptomic responses in two cancer cell lines. Transcriptome analysis revealed upregulation of mRNAs encoding cholesterol biosynthesis enzymes. This was a critical adaptive response, as a pharmacological inhibition of the pathway with statin triggered a robust apoptotic cell death accompanied by generation of a mitochondrial reactive oxygen species. The mechanistic target of rapamycin complex 1 (mTORC1), a master regulator of cell growth, is known to be engaged in controlling lipid biosynthesis. We detected the high polysomal and preribosomal peaks not only in serum-containing medium but also under serum deprivation, indicating a high rate of protein synthesis and ribosomal biogenesis independent of serum. In addition, the inhibition of mTOR kinase activity substantially reduced polysome abundance, with a more pronounced effect in serum-deprived cancer cells. Notably, the mTOR kinase inhibition also prevented the upregulation of the cholesterol synthesis enzyme that established a direct link between mTOR activity, protein synthesis, and cholesterol biosynthesis. Together, our results show that cancer cells adapt to serum withdrawal by activating the cholesterol synthesis pathway through mTOR-dependent regulation of gene expression and protein synthesis, underscoring a critical mechanism of survival under serum withdrawal.

## 1. Introduction

Cancer cells are exposed to diverse stresses in a process of tumorigenesis that includes nutrient deprivation, hypoxia, oncogenic signaling and DNA damage. All these factors challenge a cellular homeostasis. To sustain proliferation and survival, cancer cells turn on a critical adaptive mechanism by undergoing a metabolic reprogramming known as a hallmark of tumor progression [1,2,3]. Among these adaptive changes, lipid metabolism also plays an essential role with regard to the components of membranes, energy storage, and signaling molecules facilitating growth and survival [4,5]. Serum deprivation imposes severe stress by limiting both growth factors and extracellular lipids. Instead of undergoing immediate death, many cancer cells activate compensatory programs, prominently de novo lipogenesis. For example, glioblastoma multiforme cells exposed to lipid-poor conditions upregulate sterol regulatory element-binding proteins (SREBPs), which drive fatty acid and cholesterol synthesis to promote survival [6]. Similarly, breast and prostate cancer cells activate fatty acid desaturase SCD1 and other lipogenic genes in response to lipid restriction [7,8]. Withdrawal of serum is also associated with the remodeling of the lipid composition in tumor cells. Lipidomic analyses have shown that levels of triglycerides and cholesteryl esters are altered under serum-starved conditions, reflecting enhanced reliance on endogenous lipid biosynthesis and lipid storage mobilization [9]. In estrogen-receptor-positive breast cancer, resistance to estrogen deprivation has been linked to the activation of the cholesterol biosynthesis pathway, and silencing key enzymes such as MSMO1, SQLE, and EBP reduces proliferation [10]. More broadly, a restriction of exogenous lipids forces cancer cells to depend on endogenous cholesterol and fatty acid synthesis to maintain membrane integrity, redox balance, and signaling [8]. A central regulator of these anabolic processes is the mTORC1. mTORC1 integrates signals from nutrients, growth factors, and energy status to promote anabolic metabolism [11,12,13,14]. In addition to regulating protein and nucleotide synthesis, mTORC1 directly controls lipid metabolism by activating SREBP transcription factors [15,16,17]. Through this mechanism, mTORC1 stimulates the expression of genes required for fatty acid and cholesterol biosynthesis, thereby coordinating cell growth with lipid supply [18,19]. Thus, mTORC1 signaling represents a key upstream driver of the lipid metabolic programs that enable cancer cells to withstand nutrient stress. Despite these advances, the precise mechanisms by which cancer cells adapt to serum deprivation remain incompletely understood. Defining these adaptive responses is essential for uncovering metabolic vulnerabilities that may be exploited for therapeutic intervention.

## 2. Results

### 2.1. Serum Deprivation of Cancer Cells Reduces Proliferation but Does Not Induce Apoptosis

**Figure 1:**
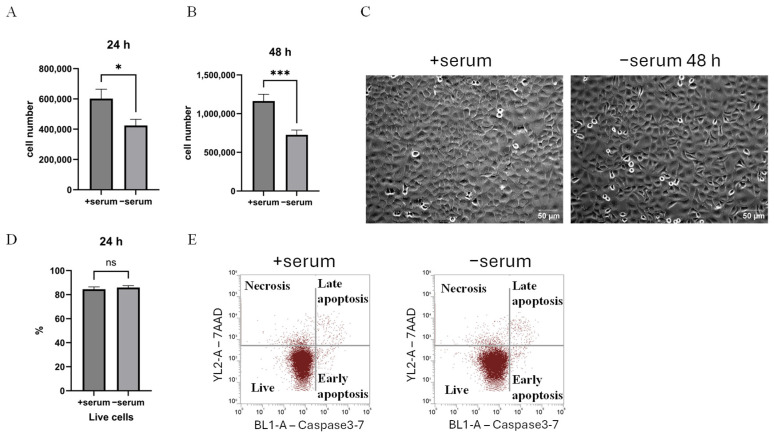
Cell proliferation but not survival is serum-dependent. H1299 cancer cell numbers grown in complete growth medium (+serum) and under serum-deprived conditions (−serum) for 24 h (**A**) and 48 h (**B**). (**A**) Cell numbers at 24 h and (**B**) at 48 h were measured using a Multisizer 4e (Beckman Coulter). Bars represent the mean ± SEM of three independent biological experiments, each performed in technical triplicates (*n* = 3). Statistical analysis was performed using Student’s *t*-test. At 24 h (**A**), the cell number under serum deprivation was significantly reduced compared to the control (+serum) (* *p* < 0.01), and at 48 h (**B**), a significant decrease was observed compared to +serum (*** *p* < 0.001). (**C**) Phase contrast images of H1299 cancer cells grown in complete growth medium (+serum) and fetal bovine serum-deprived (−serum) conditions for 48 h. In +serum, the cells have an epithelial-like morphology, and after 48 h of cell incubation in serum-deprived medium, the cells remain viable, and we did not observe cell death. These images were obtained using an inverted microscope Primo Vert microscope and a binocular phototube (Carl Zeiss, Oberkochen, Germany, article 415510-1101-000). Scale bar: 50 μm. (**D**,**E**) Flow cytometric analysis of the apoptotic response of cells upon serum deprivation using caspase 3-7 and 7-AAD staining. (**D**) Quantitative proportion of viable cells after 24 h of serum deprivation. (**E**) Dot plots showing cell populations under +serum and −serum conditions after 24 h. Data are presented as mean ± SEM from three independent biological experiments (*n* = 3). Statistical analysis was performed using Student’s *t*-test, which revealed no significant differences between +serum and −serum conditions in viable cell populations (not significant (ns)).

To examine how cancer cells adapt to growth factor deficiency, actively growing H1299 non-small cell lung carcinoma cells were switched to a serum-free cell culture condition. Within the first 24 h of serum deprivation, we observed that cancer cell numbers decreased by about 30% (Figure 1A). A more substantial decrease of about 40% in cell number was detected after 48 h of serum deprivation (Figure 1B). Importantly, the reduction in cell number was not due to cell death, as no images of detached cells were detected (Figure 1C). A flow cytometric caspase-based apoptosis assay revealed that serum deprivation did not increase apoptosis, as the proportion of apoptotic cells remained at the basal level in a range of 15%, observed in cancer cells cultured in growth medium (Figure 1D,E). Only low levels of early/late apoptotic and necrotic cells were detected that were similar in media cultures with or without serum. These findings indicate that H1299 cells adapt and survive under serum deprivation without undergoing apoptosis.

### 2.2. Transcriptomic Profiling Reveals Activation of Cholesterol Biosynthesis Pathway in Serum-Deprived Cancer Cells

**Figure 2:**
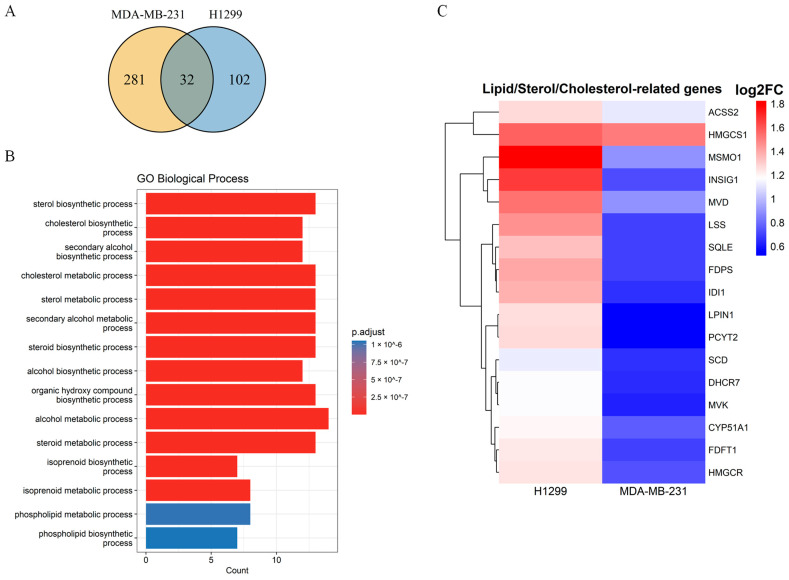
Comparative analysis of the set of upregulated genes in H1299 and MDA-MB-231 upon serum starvation. (**A**) Venn diagram shows the number of unique and common upregulated genes between H1299 and MDA-MB-231. The number of common genes that are upregulated in response to deprivation is 32. (**B**) Gene Ontology analysis of the 32 genes. Significantly enriched pathways include sterol and cholesterol biosynthesis, alcohol metabolism, and isoprenoid metabolism. Color denotes the level of statistical significance (*p*.adjust), length reflects the number of genes involved. (**C**) Heat map of genes involved in cholesterol synthesis. All analyses were performed using R (4.4.2) with the DESeq2, clusterProfiler (v4.14.6) (using org.Hs.eg.db (v3.20.0)), pheatmap, and VennDiagram packages. Data were imported/exported with readxl (v1.4.5) and openxlsx (v4.2.8).

To characterize adaptive responses, we performed total RNA sequencing from H1299 and MDA-MB-231 cells cultured under serum starvation for 24 h. Triplicate libraries were sequenced on an Illumina NovaSeq 6000 platform, and differentially expressed genes (DEGs) were defined as transcripts with ≥2-fold expression change. To define the adaptive response to the serum deprivation of cancer cells, the upregulation pathways were identified by Gene Ontology (GO) analysis. A comparative analysis identified upregulated 102 DEGs in serum-deprived H1299 cells. In the same setting, a higher number of the DEGs (281) were found in MDA-MB-231 cells (Figure 2A), with the distinctive increase in the gene transcripts belonging to the HIF-1 signaling, glycolysis/gluconeogenesis, carbon metabolism, and pentose phosphate pathways (Supplementary Figure S1A). Most of the identified upregulated DEGs were cell type-specific and only 32 DEGs were shared in both cell lines (Figure 2A). The GO analysis of these shared upregulated DEGs identified in both cancer cell lines revealed that these transcripts encode the sterol and cholesterol biosynthesis pathway components (Figure 2B). In addition, the heat maps of cholesterol-related transcripts demonstrated a substantial upregulation of the transcripts engaged in cholesterol synthesis, including the key mevalonate pathway components *HMGCS1* and *MVD* in both cell types (Figure 2C). The serum-dependent transcriptomic study of the two non-related (lung and breast) cancer cell lines was informative in identifying a common response to serum deprivation.

**Figure 3:**
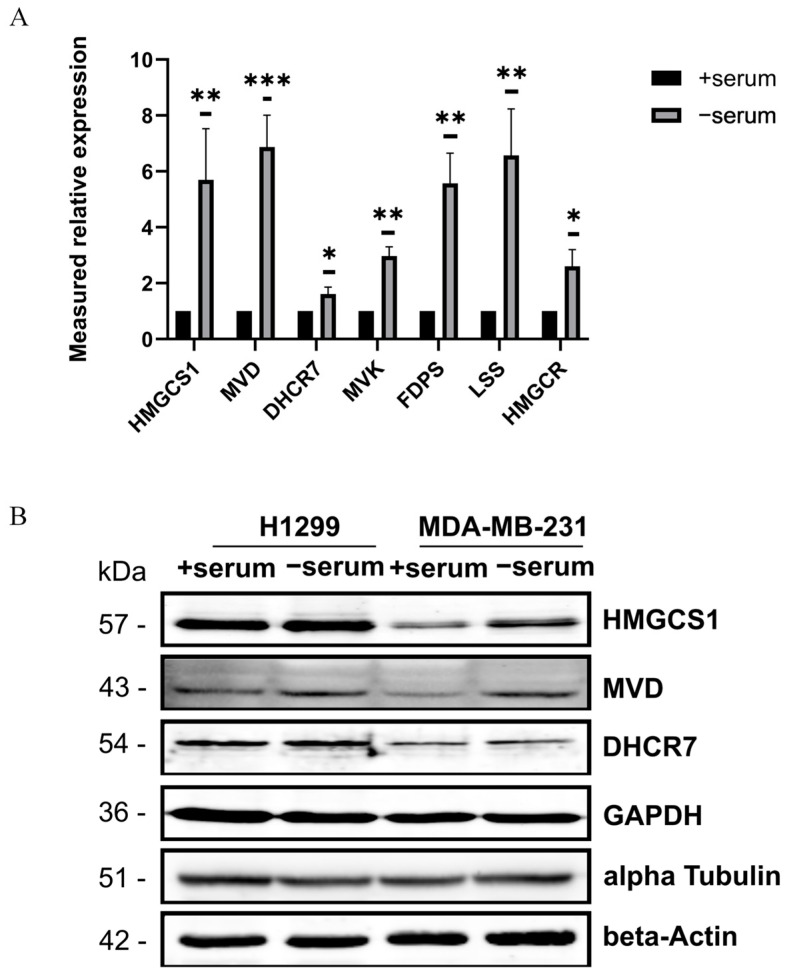
Expression of the mevalonate pathway components in cancer cells is serum-dependent. (**A**) Relative expression (normalized to GAPDH) of mevalonate pathway genes (HMGCS1, MVD, DHCR7, MVK, FDPS, LSS, HMGCR) in H1299 cells grown with (+serum) and without serum (−serum). Data are presented as mean ± SEM (*n* = 3), *p* < 0.05 (*), *p* < 0.01 (**), *p* < 0.001 (***), calculated using Student’s *t*-test. (**B**) Immunoblotting analysis showing expression of mevalonate and sterol pathway proteins in serum-deprived H1299 and MDA-MB-231 cells. Protein levels of HMGCS1, MVD, and DHCR7 were assessed in the lysates of H1299 and MDA-MB-231 cancer cells incubated in complete medium (+serum) or serum-deprived medium (−serum) for 24 h. GAPDH, β-Actin, and α-Tubulin were detected as loading controls.

To validate the transcriptional activation of the mevalonate pathway, we performed quantitative PCR (qPCR) analysis of seven key enzymes of the pathway engaged in cholesterol and isoprenoid biosynthesis, including 3-hydroxy-3-methylglutaryl-CoA synthase 1 (HMGCS1) [20], mevalonate diphosphate decarboxylase (MVD) [21], 7-dehydrocholesterol reductase (DHCR7) [22], mevalonate kinase (MVK) [23], farnesyl diphosphate synthase (FDPS) [24], lanosterol synthase (LSS) [25], and 3-hydroxy-3-methylglutaryl-CoA reductase (HMGCR) [26]. The qPCR results demonstrated a consistent upregulation of all examined genes with the differences in fold increase. The strongest increases were observed for MVD and LSS (6-fold) and HMGCS1 and FDPS (5-fold), while HMGCR and MVK displayed a more moderate 2-fold increase, and DHCR7 showed the lowest induction (1.6-fold) (Figure 3A). A coordinated elevation across all seven transcripts in the mevalonate pathway indicates a pathway activation rather than an isolated gene-specific effect.

To further corroborate the transcriptomic findings, we assessed protein abundance by immunoblotting. The cellular lysates from H1299 and MDA-MB-231 cancer cells subjected to 24 h serum deprivation exhibited about a two-fold increase in abundance of HMGCS1 and MVD proteins compared with cells cultured with serum (Figure 3B). In contrast, only a marginal increase in the DHCR7 protein was detected (Figure 3B), consistent with the modest transcript upregulation observed by qPCR (Figure 3A). Together, our results demonstrate that serum deprivation activates metabolic programs promoting lipid and sterol biosynthesis that might compensate for the absence of exogenous lipids normally supplied by serum.

### 2.3. Simvastatin-Induced Apoptosis in Serum-Deprived Cancer Cells Mediated by a Mitochondrial ROS Generation

**Figure 4:**
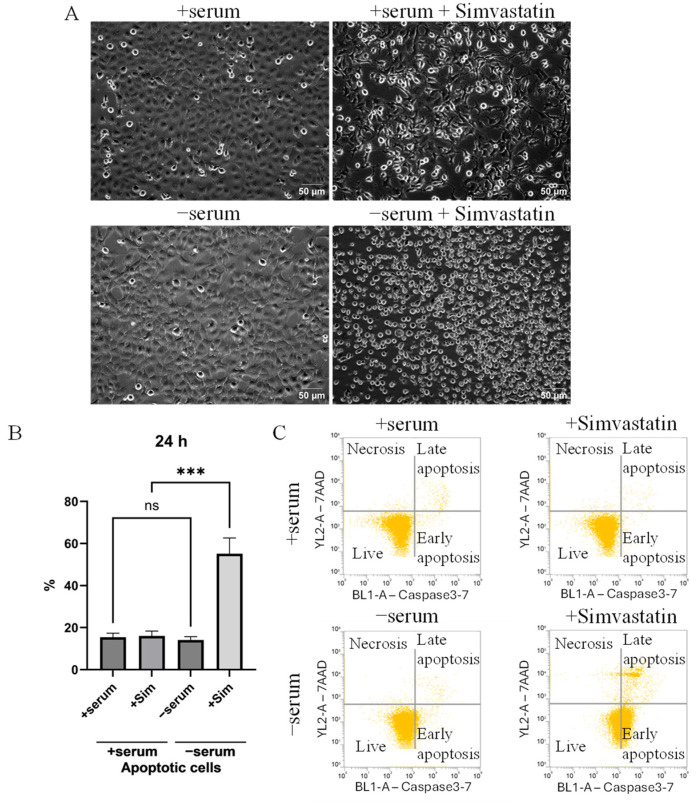
Prevention of the cholesterol synthesis in serum-deprived cancer cells induced an apoptotic response. (**A**) Morphology of H1299 cells treated with Simvastatin. Cancer cells were grown in complete medium (+serum) or without serum (−serum) in the presence or absence of Simvastatin (20 μM, 24 h). With +serum + Simvastatin, cellular stress occurs, which is noticeable in the change in morphology, but with −serum + Simvastatin, cell death is observed, accompanied by the detachment of cells from the plate. These images were obtained using an inverted microscope Primo Vert microscope stand with a binocular phototube (Carl Zeiss, article 415510-1101-000). (**B**,**C**) Apoptotic assay using caspase 3-7 and 7-AAD, conducted via flow cytometry. (**B**) Bar graph illustrates the effect of Simvastatin (+Sim, cholesterol synthesis inhibitor) under normal serum (+serum) and serum-deprived (−serum) conditions. Graph represents the distribution of non-viable cells, which include early and late apoptosis and necrosis combined. Simvastatin treatment strongly increases the non-viable cell population under serum-deprived conditions. Bars represent the mean ± SEM of three independent biological experiments (*n* = 3). Statistical analysis was performed using Ordinary one-way ANOVA. *p* < 0.001 (***) and ns indicates not significant. (**C**) Representative flow cytometry dot plots showing caspase 3–7 activation (BL1-A) and 7-AAD staining (YL2-A). Quadrants show the percentage of live, early apoptotic, late apoptotic, and dead cells. There is a strong shift toward apoptotic populations in the −serum + Simvastatin condition compared to +serum + Simvastatin.

To test the functional relevance of lipid biosynthesis under serum deprivation, we inhibited cholesterol synthesis, applying its specific inhibitor simvastatin by treating cancer cells with a dose of 20 μM. Simvastatin treatment markedly increased apoptotic cell death in serum-deprived cancer cells, according to the appearance of detached round cells (Figure 4A) and the results of a caspase-3/7 activation and 7-AAD staining assay (Figure 4B,C). Quantification confirmed a significant reduction in viable cells and the strong induction of apoptotic cells in a range of 40% (serum-deprived + Simvastatin). By contrast, simvastatin did not show a cytotoxic effect in cancer cells cultured in a serum-containing medium. These results indicate that a cholesterol synthesis pathway is essential for cancer cell survival under serum withdrawal.

A high cytotoxic impact of Simvastatin in serum-deprived conditions was observed in H1299 cancer cells, suggesting that malignant cells are sensitive to combined metabolic stress. To assess whether this response is also evident in non-tumorigenic cells, similar experiments were conducted using the immortalized keratinocyte cell line HaCaT. Morphologically, HaCaT cells maintained their typical epithelial-like appearance, indicating the absence of significant cytotoxicity even under the combined treatment of Simvastatin and serum starvation (Supplementary Figure S2A). Consistently, flow cytometric analysis of caspase-3/7 activity together with 7-AAD staining revealed no substantial increase in the proportion of dead cells, with cell death remaining at approximately 25% across all conditions (Supplementary Figure S2B). These findings suggest that malignancy is associated with an enhanced susceptibility to cholesterol synthesis inhibition under serum-deprived conditions.

**Figure 5:**
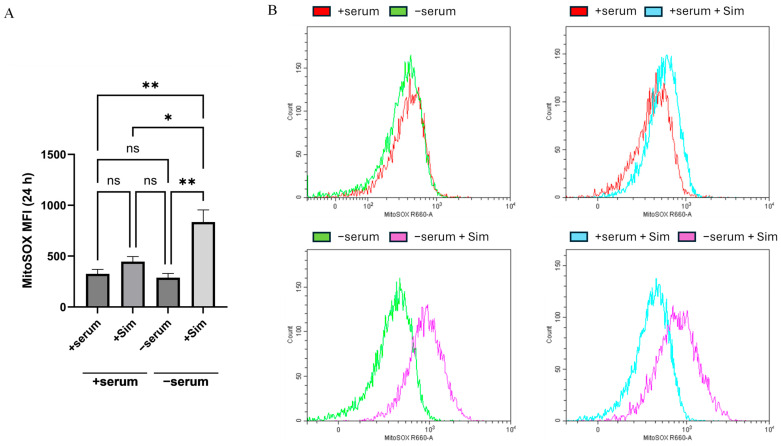
An apoptotic response provoked by Simvastatin treatment of serum-deprived cancer cells is accompanied by the generation of a mitochondrial reactive oxygen species (ROS). (**A**) The graph illustrates the mean fluorescence intensity (MFI) of MitoSOX^TM^ dye after 24 h of culture of H1299 cells under serum (+serum), serum-deprived (−serum), and Simvastatin (+Sim) conditions. Serum starvation with Simvastatin (20 uM) significantly increased mitochondrial ROS, *p* < 0.05 (*), *p* < 0.01 (**). Bars represent the mean ± SEM of three independent biological experiments (*n* = 3). Statistical analysis was performed using Ordinary one-way ANOVA. *p* < 0.05 (*), *p* < 0.01 (**), and ns indicates not significant. (**B**) Histograms of fluorescence distribution (R660-A channel) show a rightward shift in the signal for “−serum + Simvastatin” compared to “+serum + Sim” and “−serum”. This confirms the increase in ROS. To detect ROS, we used MitoSOX^TM^ Red (Invitrogen, Thermo Fisher Scientific, Waltham, MA, USA; Cat# M36008), a dye that reacts with superoxide in mitochondria. Our results demonstrate that Simvastatin treatment increases oxidative stress in growth factor deficiency.

Since inhibition of cholesterol synthesis can induce oxidative stress, we next examined mitochondrial reactive oxygen species (ROS). ROS levels were measured using MitoSOX Red in H1299 cancer cells. Simvastatin treatment of serum-deprived cancer cells significantly increased mitochondrial ROS detection compared to all other conditions (Figure 5A,B). Flow cytometric histograms indicated a substantial increase in ROS-producing cancer cells in serum-deprived cells treated with simvastatin (mean fluorescence intensity about 1.9-fold). Thus, simvastatin promotes the generation of mitochondrial ROS specifically under serum-deprived conditions, which links cholesterol biosynthesis inhibition to oxidative stress-induced cytotoxicity.

To validate the role of reactive oxygen species (ROS) in simvastatin-induced apoptosis in serum-deprived cancer cells, an experiment was conducted using the antioxidant N-acetyl-L-cysteine (NAC). After replacing the medium with a serum-free medium, NAC was added at a final concentration of 5 mM, and after 1 h, simvastatin was added at a final concentration of 20 μM. Next, cell viability and caspase-3/7 activity with 7-AAD were assessed using flow cytometry. The results of the experiment showed that NAC treatment prevented the decrease in viability and accumulation of apoptotic cells (Supplementary Figure S3A,B) observed with simvastatin under serum deprivation conditions. These results demonstrate that the generation of ROS is a key factor mediating apoptotic cell death induced by simvastatin in serum-deprived cancer cells.

### 2.4. The Serum-Dependent Adaptive Upregulation of a Cholesterol Synthesis Pathway Is Mediated by mTOR Kinase Activity

We show that cancer cells adapt to serum deprivation by upregulation of the mRNAs encoding enzymes engaged in cholesterol synthesis. This critical adaptive mechanism carries out a serum-dependent transcriptional regulation of the mevalonate pathway, allowing cancer cells to sustain cholesterol and isoprenoid production required for membrane integrity, lipid raft mediated signaling, and protein prenylation. Previous studies have demonstrated that mTORC1 signaling acts as a master regulator of lipid metabolism, including cholesterol synthesis, through transcriptional activation of SREBP2 and its downstream target genes. Based on these findings, we investigated whether the serum-dependent activation of cholesterol synthesis is mediated by mTORC1 signaling.

**Figure 6:**
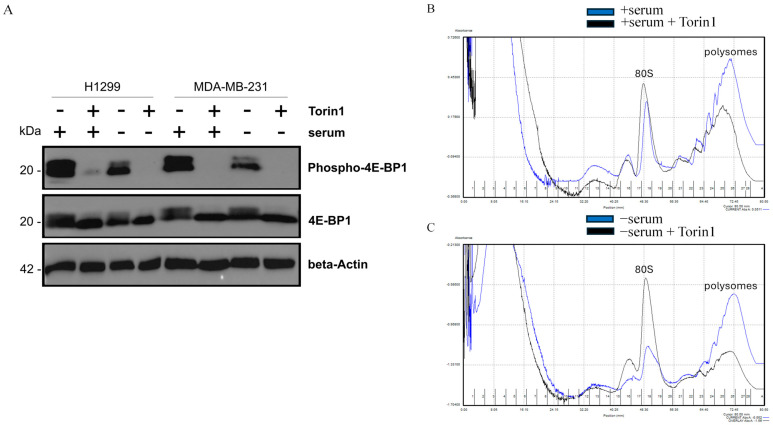
The serum-dependent mTORC1 signaling and polysome profiling. (**A**) Immunoblotting shows inhibition of 4E-BP1 phosphorylation by Torin1 treatment in H1299 and MDA-MB-231. Cells were treated with the mTORC1 inhibitor Torin1 (250 nM) for 24 h in +serum and −serum. To assess mTORC1 activity, antibodies to the phosphorylated form of 4E-BP1 (P-4E-BP1) were used. Torin1 reduced phospho-4E-BP1 levels in all cells regardless of the presence of serum, indicating inhibition of mTOR signaling. Total 4E-BP1 protein abundance was similar in all conditions but its mobility was mTOR-dependent. Beta-actin was used as a protein loading control. (**B**,**C**) The effects of Torin1 on polysomes and 80S ribosomes in control (+serum) and −serum cell culture conditions. Profiles were obtained using the Biocomp gradient station. Profile for +serum cell culture condition (**B**) and −serum cell culture condition (**C**). The blue line represents profiles for H1299 cancer cells grown in serum-deprived and +serum conditions, and the black line shows the profile after treatment with 250 nM Torin1 for 24 h. Torin1 reduces polysomal peaks and increases 80S ribosome peaks, reflecting translational inhibition.

To assess mTORC1 activity under serum deprivation, we examined the phosphorylation status of the mTORC1 substrate 4E-BP1 by immunoblotting with a phospho-specific antibody [27]. Serum-deprived H1299 and MDA-MB-231 cells exhibited only a partial reduction in a range of 30% in 4E-BP1 phosphorylation, whereas treatment with the mTOR kinase inhibitor Torin 1 fully suppressed 4E-BP1 phosphorylation irrespective of serum availability (Figure 6A). These findings indicate that mTORC1 retains its basal activity in serum-deprived cancer cells, suggesting the maintenance of mTORC1-dependent protein synthesis.

To further characterize a serum-dependent translational capacity, we performed the polysome and preribosomal profiles by sucrose gradient fractionation of the cancer cell and nuclear lysates obtained from cells incubated with or without serum as previously described [28]. After 24 h of serum deprivation, the abundance of polysomes and preribosomes was comparable to that of serum-fed cancer cells, while a modest non-significant decrease in polysomes of about 20% was observed after 48 h (Supplementary Figure S4A–C). Interestingly, the preribosomal peaks were similar or at the same height in serum-fed or starved cancer cells for 48 h. These data indicate that protein synthesis remains robust for at least 24 h in serum-deprived cells, accompanied by the rate of ribosomal biogenesis detected in actively growing cancer cells. The treatment of cancer cells with Torin 1 [29] substantially reduced polysome levels, reaching a decrease of 27% in serum-fed cells, while a more pronounced 51% decrease was observed in serum-deprived cancer cells. The Torin 1 effect in serum-deprived cells was accompanied by a marked accumulation of an 80S ribosome peak that was increased two-fold, compared with only a minor increase of about 15% in serum-fed cells. Together, these results demonstrate that cancer cells maintain active mTORC1-dependent protein synthesis under serum withdrawal.

**Figure 7:**
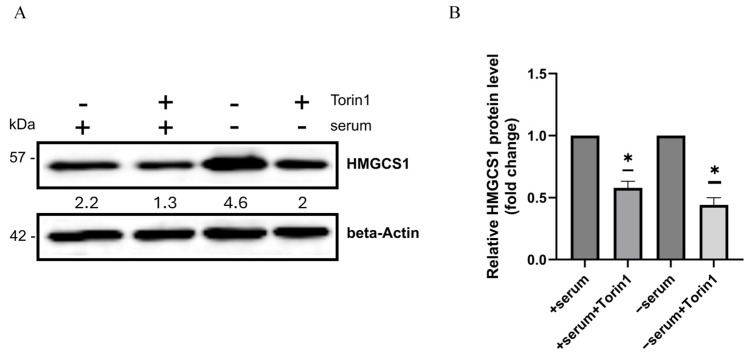
The upregulation of HMGCS1 protein abundance in serum deprivation is mTOR-dependent. (**A**) The cellular lysates obtained from H1299 cancer cells treated with or without Torin1 (250 nM) for 24 h in +serum or serum conditions were analyzed by immunoblotting with the HMGCS1 antibody. β-Actin was used as a loading control. (**B**) Quantification of HMGCS1 levels as fold change. Data are presented as mean ± SEM (*n* = 3), *p* < 0.05 (*), calculated using one sample *t*-test. There was a significant decrease in HMGCS1 protein abundance in H1299 upon serum deprivation with Torin1 treatment.

Finally, to directly link mTORC1 signaling with transcriptional upregulation of the cholesterol synthesis pathway, we examined HMGCS1 expression in H1299 cells treated with Torin 1. The immunoblotting study indicates that upregulation of the mevalonate pathway enzyme HMGCS1 in serum-deprived cancer cells was prevented by the mTOR kinase inhibitor Torin 1 (Figure 7). This finding links the mTORC1 kinase signaling turning on expression of the mevalonate pathway enzyme in the adaptative response of cancer cells to serum withdrawal.

## 3. Discussion

Our study reveals a critical adaptive mechanism that enables cancer cells to survive under serum-deprived conditions. We show that serum deprivation induces a transcriptional upregulation of the cholesterol/mevalonate synthesis pathway, which provides critical metabolic support for cell viability. Inhibition of this pathway with statins triggered apoptotic cell death accompanied by a robust increase in ROS, highlighting the essential role of cholesterol and isoprenoid biosynthesis in serum-deprived cancer cells to maintain redox homeostasis and cell survival.

Our data show that serum deprivation induces a coordinated but heterogeneous upregulation of mevalonate pathway genes in cancer cells. Enzymes at key nodes such as MVD, LSS, HMGCS1, and FDPS were strongly induced, while HMGCR, MVK, and DHCR7 showed only modest increases. This pattern suggests a selective enhancement of steps required for generating sterol intermediates and isoprenoids, rather than uniformly boosting cholesterol production. It is possible that mTORC1–SREBP2 signaling drives this response, with additional post-transcriptional control over HMGCR. Such rewiring creates a metabolic dependency: inhibition of the pathway with statins disrupts isoprenoid supply and triggers apoptosis, highlighting a therapeutic vulnerability under nutrient stress.

Mitochondria are the major source of ROS in cancer cells and the precise mechanisms by which ROS are generated remain incompletely understood. An electron leakage from complexes I and III of the mitochondrial electron transport chain is likely an explanation [30,31]. A metabolic rewiring in cancer cells, altered redox buffering, and changes in the lipid composition of mitochondrial membranes likely modulate ROS output in more complex ways [32,33]. The disruption of cholesterol and isoprenoid metabolism may impair mitochondrial function by altering membrane integrity, electron carrier dynamics, or the prenylation of proteins involved in mitochondrial homeostasis [34,35]. Our data suggest that the disruption of cholesterol and isoprenoid metabolism may impair mitochondrial integrity and function, thereby exacerbating electron leakage and ROS production. Thus, while our results firmly establish a correlation between the blockade of cholesterol synthesis and ROS accumulation, the mechanistic basis of mitochondrial ROS generation in cancer cells requires further study.

We also demonstrate that the adaptive upregulation of cholesterol synthesis depends on mTORC1 signaling. Our data indicate that the observed adaptive response of cancer cells to serum deprivation is carried out by a high capacity of ribosomal biogenesis and protein synthesis. Interestingly, serum deprivation only partially suppressed mTORC1 activity, indicating that residual signaling is sufficient to drive SREBP2-dependent transcription of cholesterol synthesis genes. Treatment with an ATP-competitive inhibitor Torin 1 fully abrogated this adaptive response, confirming that mTORC1 is a central regulator of cholesterol synthesis under serum-deprived stress conditions. These findings extend previous reports of mTORC1-dependent SREBP2 regulation by showing that this axis is specifically engaged to sustain survival under serum limitation.

An unexpected observation from our study was that the polysomal peaks of cancer cells were more sensitive to Torin 1 under serum deprivation than under serum-supplied conditions. This suggests that residual mTORC1 signaling sustains not only cholesterol synthesis but also active protein translation under stress. The disproportionate loss of polysomes upon Torin 1 treatment implies that cancer cells rely on mTORC1 to preserve a selective translational program that supports a metabolic adaptation under serum deprivation. One possibility is that mRNAs encoding cholesterol synthesis enzymes or regulators of SREBP2 activity are preferentially maintained under these conditions. Thus, a master regulator of cell growth mTORC1 may act as a hub coordinating the adaptive lipid synthesis and active protein synthesis to ensure survival during serum deprivation.

Taken together, our findings indicate that cancer cells prioritize cholesterol synthesis when challenged with serum deprivation and that this adaptation is tightly controlled by residual mTORC1 signaling. Blocking cholesterol synthesis or inhibiting mTOR kinase activity disrupts this adaptive program, leading to impaired polysome maintenance, excessive ROS accumulation, and apoptotic cell death. These results not only highlight a metabolic vulnerability that could be therapeutically exploited but also suggest potential benefits of combining mTOR inhibitors with statins to target nutrient-stressed tumor cells.

Future work should explore the mechanistic link between cholesterol metabolism and mitochondrial ROS production, as well as the specific translational programs preserved by residual mTORC1 signaling under stress of serum withdrawal. Addressing these questions will clarify how metabolic and translational rewiring cooperatively promote cancer cell survival and may guide the development of combined therapeutic strategies targeting both pathways.

## 4. Materials and Methods

### 4.1. Cell Culture

H1299 (human non–small cell lung cancer) [36] and MDA-MB-231 (triple-negative breast cancer, TNBC) [37] cell lines were obtained from ATCC (Manassas, VA, USA). Cells were cultured in Dulbecco’s modified Eagle’s medium (DMEM)/F-12 (US Biological Life Sciences, Salem, MA, USA; Cat# D9807) supplemented with 10% fetal bovine serum (FBS) (Gibco™, Thermo Fisher Scientific, Waltham, MA, USA; Cat# A3160802), 2 mM glutamine (Sigma-Aldrich, St. Louis, MO, USA; Cat# G3126), 100 U/mL penicillin (Sigma-Aldrich, Cat# P3032), and 0.1 mg/mL streptomycin (Sigma-Aldrich, St. Louis, MO, USA; Cat# S9137) at 37 °C in a humidified incubator with 5% CO_2_. For serum deprivation experiments, the same DMEM/F-12 medium without FBS was used. For subcellular fractionation and cytoplasmic extract preparation, 5 × 10^6^ H1299 cells were seeded in 145 mm dishes with 20 mL culture medium and grown for 24 h and 48 h.

### 4.2. Cell Counting and Size Analysis

Culture medium was collected into a conical tube, and adherent cells were washed with PBS, detached with 1× Trypsin (Capricorn Scientific, Ebsdorfergrund, Germany; Cat# TRY-1B10), and pooled with the medium and PBS wash. Cell number and size distributions were determined using a Multisizer 4e Coulter Counter (Beckman Coulter, Brea, CA, USA) [38].

### 4.3. RNA Isolation, Library Preparation, and Sequencing

Total RNA was isolated using the mirVana™ miRNA Isolation Kit (Invitrogen™, Thermo Fisher Scientific, Waltham, MA, USA; Cat# AM1560) following the manufacturer’s protocol. RNA concentration and purity were assessed using a NanoDrop 2000c (Thermo Fisher Scientific, Waltham, MA, USA) and a Qubit 4 Fluorometer (Thermo Fisher Scientific, Waltham, MA, USA), and RNA integrity was evaluated using an Agilent 2100 Bioanalyzer (Agilent Technologies, Santa Clara, CA, USA). Libraries were prepared with the Illumina Stranded Total RNA Prep kit with rRNA depletion (Ribo-Zero Plus, Illumina, San Diego, CA, USA; Cat# 20040529). After rRNA removal, RNA was fragmented, reverse-transcribed into cDNA, ligated with adapters, and PCR amplified. Libraries were quality-checked and sequenced on an Illumina NovaSeq 6000 (Illumina, San Diego, CA, USA) platform to generate paired-end 2 × 100 bp reads [39].

### 4.4. Transcriptomic and Pathway Analysis

Raw sequencing reads from NovaSeq 6000 NGS platforms were demultiplexed using the bcl2fastq tool (Illumina, San Diego, CA, USA). RNA-seq reads were quality-checked with FastQC and summarized using MultiQC (v1.14). Alignment and quantification were performed with the Illumina Dragen RNA pipeline (v4.3.6) against the hg19 reference genome, producing transcript-level quantifications and gene counts. Count matrices were assembled using tximport and low-count features were filtered. Differential expression was analyzed in R (v4.4.2) via DESeq2, using an in-house R script as the analytical backbone. The detailed bioinformatics pipeline is available via GitHub (https://github.com/LabBandSB/DESeq2-RNASeq-Pipeline, accessed on 30 September 2025). Gene symbols were mapped to Entrez IDs using org.Hs.eg.db (v3.20.0). Pathway enrichment analysis was performed with clusterProfiler (v4.14.6) using Gene Ontology (Biological Process (BP) categories; pAdjustMethod = “BH”; *p* < 0.05). Enrichments were visualized with enrichplot (v1.26.6) (dotplot, showCategory = 20). Genes were classified by thresholds of |log_2_FC| > 0.5 or 1 and padj < 0.05. Heatmaps for cholesterol-related genes were generated using pheatmap (row clustering only). Results were exported to Excel (openxlsx (v4.2.8)).

### 4.5. Flow Cytometry

For caspase-3/7 activity H1299 and HaCaT cells were seeded in six-well plates and cultured for 24 h before replacing growth medium with serum-free medium. Cells were treated with 20 μM simvastatin (Supelko, Sigma-Aldrich, St. Louis, MO, USA; Cat# PHR1438) in either complete medium or serum-free medium for 24 h. Cells were stained with Caspase-3/7 Green reagent (CellEvent Kit; Thermo Fisher Scientific, Waltham, MA, USA; Cat# C10427) for 25 min at 37 °C, followed by SYTOX AADvanced Dead Cell Stain (Thermo Fisher Scientific, Cat# C10427) for 5 min. Cells were analyzed on an Attune NxT flow cytometer (Thermo Fisher Scientific). Caspase-3/7^+^/SYTOX^−^ cells were considered early apoptotic, while Caspase-3/7^+^/SYTOX^+^ cells were late apoptotic.

After 24 h of cell seeding, the medium was changed to serum-free medium. Immediately after the medium change, N-acetyl-L-cysteine (NAC) (Sigma-Aldrich, Cat# A7250) was added at a final concentration of 5 mM. Simvastatin was then added at a final concentration of 20 μM after one hour. After 24 h, the cells were analyzed using flow cytometry. Cells growing in six-well plates were harvested: the medium from each well was transferred to a separate 15 mL tube, the cells were washed with 1× PBS, trypsin was added, and the cells were collected and pelleted by centrifugation. Cell pellets were stained with CellEvent™ Caspase-3/7 Green Detection Reagent (Thermo Fisher Scientific, Cat# C10427) at 37 °C for 25 min and SYTOX™ AADvanced™ Dead Cell Stain (Thermo Fisher Scientific, Cat# C10427) for 5 min, also at 37 °C.

### 4.6. Mitochondrial ROS Measurement

Mitochondrial ROS were measured using MitoSOX™ Red (Invitrogen, Thermo Fisher Scientific, Waltham, MA, USA; Cat# M36008). H1299 cells were treated with 20 μM simvastatin in either complete or serum-free medium for 24 h. Cells were harvested, washed with PBS, and resuspended in HBSS containing MitoSOX Red. After 30 min incubation at 37 °C, cells were washed with HBSS (Gibco, Thermo Fisher Scientific, Waltham, MA, USA; Cat# 14175-046) and immediately analyzed by flow cytometry (CytoFLEX S, Beckman Coulter, Brea, CA, USA), as previously described [40].

### 4.7. Quantitative Real-Time PCR

Quantitative real-time PCR (qPCR) was performed using gene-specific primers. GAPDH was used as an endogenous control. Two negative controls were used to monitor reaction quality: one without template DNA (no cDNA) and one without the reverse transcription step (no RT). Relative gene expression levels were calculated using the comparative 2^−ΔΔCt^ method.

### 4.8. Immunoblotting

Cells were lysed in 1× RIPA buffer (20 mM Tris-HCl pH 7.5, 150 mM NaCl, 1 mM EDTA, 1 mM EGTA, 1% NP-40, 0.5% sodium deoxycholate) supplemented with protease and phosphatase inhibitors (Thermo Fisher Scientific, Cat# 78442). Protein concentration was determined by the Bradford method. Equal amounts of protein were resolved by SDS-PAGE (10% or 15% gels) and transferred to PVDF membranes (Millipore, Burlington, MA, USA; Cat# IPVH00010). Membranes were blocked with 5% milk or 5% BSA in PBST (0.1% Tween-20) for 1 h, then incubated overnight at 4 °C with primary antibodies against phospho-4E-BP1 (1:1000, CST, 2855), 4E-BP1 (1:1000, CST, 9644), HMGCS1 (1:1000, CST, 42201), MVD (1:500, Abcam, ab129061), DHCR7 (1:1000, Abcam, ab103296), β-actin (1:1000, CST, 4970), α-Tubulin (1:2000, Abcam, Cambridge, UK; ab7291), and GAPDH (1:1000, CST, 2118). After washing, membranes were incubated with HRP-conjugated (1:10,000, Cusabio, Wuhan, China; Cat# CSB-PA564648 or CSB-PA573747) or IRDye-conjugated secondary antibodies (1:10,000, LI-COR, Cat# 926-68071 or 926-32210) for 1–2 h at room temperature. Signals were detected using Clarity ECL substrate (Bio-Rad, Hercules, CA, USA; Cat# 170-5061) on a ChemiDoc system (Bio-Rad) or with infrared detection (LI-COR Odyssey CLx). Densitometry was performed using Image Lab (Bio-Rad) or Image Studio (LI-COR). Protein expression was normalized to β-actin, GAPDH, or α-Tubulin.

### 4.9. Subcellular Fractionation to Obtain the Soluble Nuclear Fractions and Cytoplasmic Fractions from Cancer Cells

Every step was carried out at 4 °C. Twenty mL of cold PBS was used to wash the cells. Washed cells were lysed in Magnesium (Mg) Buffer that was supplemented with the Halt^TM^Protease/Phosphatase Single-Use Inhibitor Cocktail (Thermo Fisher Scientific, Cat# 78442). Mg Buffer was composed of 40 mM Hepes-NaOH, pH 7.5, 160 mM KCl, 10 mM MgCl_2_, 0.5% glycerol, and 0.5% Nonidet P-40. H1299 cells from the following conditions were used to obtain cytoplasmic fractions: +serum (control), +serum + Torin1, −serum, and −serum + Torin1. Torin1 (Sigma-Aldrich, Cat# 475991) at a final concentration of 250 nM was incubated for 24 h. Cells from each plate (one for each condition) were scraped into 2 mL tubes and then incubated for 30 min with rotation in a cold room. Following the incubation period, the lysates were spun for five minutes at 500× *g* to separate the cytoplasm (supernatant) and nuclei (pellet). One milliliter of detergent-free Mg Buffer was used to wash the nuclei, which were then resuspended by flicking and spinning at 500× *g* for 5 min, with the supernatant being discarded. The nuclei were further cleaned by adding 0.4 mL of Nuclear Lysis Buffer which has the following contents: 10 mM Tris-HCl, pH 8.0, 2.5 mM MgCl_2_, 1.5 mM KCl, 0.5% Triton X-100, and 0.5% deoxycholate (prior to use, this buffer was supplemented with MgCl_2_, the initial stock was prepared without MgCl_2_; does not contain protease or phosphatase inhibitors) and incubating with rotation for 15 min in a cool room. This rigorous washing step was added in order to perforate the nuclear envelope and eliminate any remaining cytoplasmic elements, including mitochondria. Following the stringent washing, nuclei were collected by spinning at 500× *g* for five minutes and washed again with 1 mL of Mg Buffer in the absence of detergent. The nuclei were collected in one tube by spinning at 500× *g* for 5 min. The nuclei pellets that were mixed in one tube were lysed in 0.4 mL of Nuclear Lysis Buffer (with Halt^TM^Protease/Phosphatase Single-Use Inhibitor Cocktail). The obtained nuclei were subjected to four cycles of 15 s of sonication followed by a 30 s pause using a Bioruptor (Diagenode) to achieve mild nuclear lysis. The nuclear lysates were spun for 15 min at 20,000× *g* and 4 °C to obtain the soluble nuclear fraction after the sonication step [28].

### 4.10. Sucrose Gradient Fractionation

A 0–60% linear sucrose gradient tube had about 1–3 mg of cytoplasmic and soluble nuclear fraction loaded on top of it. An ultracentrifugation tube (Seton) with 0% sucrose at the bottom was filled with an equal volume of 60% sucrose to create the sucrose gradients in Sucrose Gradient Buffer, which contains 20 mM Hepes, pH 7.5, 100 mM KCl, and 5 mM MgCl_2_. Obtaining highly reproducible linear sucrose gradients and performing automated fractionation analysis were made possible by the BioComp gradient station equipped with Triax flow cell 1 from BioComp Instruments (Fredericton, NB, Canada). A ThermoFisher Sorvall^TM^ ultracentrifuge with the SW41 Rotor type was used to separate the cytoplasmic and soluble nuclear fraction samples. The centrifuge was set to accelerate at 9 and decelerate at 4 for 3 h and 15 min at 4 °C. After ultracentrifugation, the BioComp gradient station with Triax flow cell 1 fractionated the sucrose gradients, yielding 28 fractions, by measuring UV light absorbance (254 nm wavelength) to identify ribosomal, polysomal, and preribosomal complexes.

### 4.11. Statistical Analysis

Data were analyzed using GraphPad Prism v10. Statistical significance was determined using Student’s *t*-test or one-way ANOVA as appropriate. A *p*-value < 0.05 was considered significant.

## Data Availability

All sequence data presented in this study are deposited in the NCBI Sequence Read Archive (SRA) repository and are publicly available under accession BioProject number PRJNA1337462 (https://www.ncbi.nlm.nih.gov/bioproject/?term=(PRJNA1337462), accessed on 9 October 2025). Count matrixes with differentially expressed genes after bioinformatics processing of RNA-seq data from this study can be provided upon request.

## Supplementary Materials

The following supporting information can be downloaded at https://www.mdpi.com/article/10.3390/ijms262210932/s1.

## Abbreviations

The following abbreviations are used in this manuscript:

mTOR	mechanistic target of rapamycin
HMGCS1	3-hydroxy-3-methylglutaryl-CoA synthase 1
MVD	mevalonate diphosphate decarboxylase
DHCR7	7-dehydrocholesterol reductase
MVK	mevalonate kinase
FDPS	farnesyl diphosphate synthase
LSS	lanosterol synthase
HMGCR	3-hydroxy-3-methylglutaryl-CoA reductase
