# Supplementary material for: The Adaptation of Cancer Cells to Serum Deprivation Is Mediated by mTOR-Dependent Cholesterol Synthesis
Source: Int J Mol Sci. 2025 Nov 12;26(22):10932. doi: 10.3390/ijms262210932 (PMC12652518; doi:10.3390/ijms262210932)
Supplement: Supplementary file 1 [file ijms-26-10932-s001.zip › ijms-3934435-supplementary.pdf]

Supplementary materials

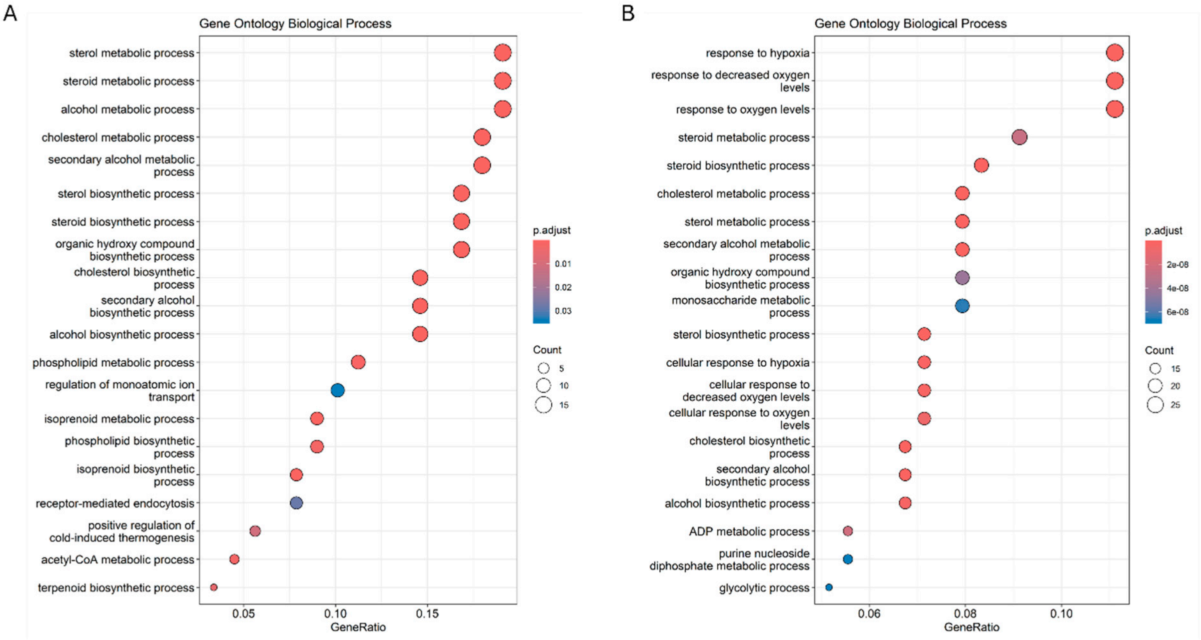

Supplementary Figure 1. (A) Functional transcript analysis of H1299 cells after 24 h of serum starvation. Gene Ontology term enrichment analysis for the biological processes of the upregulated genes. The major pathways are sterol and cholesterol metabolism and biosynthesis. Circle sizes represent gene counts and colors represent p.adjust. (B) Functional transcriptomic analysis of MDA-MB-231 cells after 24 h of serum starvation. Gene Ontology term enrichment analysis for the biological processes of upregulated genes. The top pathways are cholesterol metabolic process, sterol biosynthetic process, steroid metabolic process, response to hypoxia and response to decreased oxygen levels. Circle size represents gene counts and color represents p.adjust. All analyses were performed in R (4.4.2), differential expression was computed with DESeq2; GO enrichment with clusterProfiler (using org.Hs.eg.db); visualization with enrichplot/ggplot2.

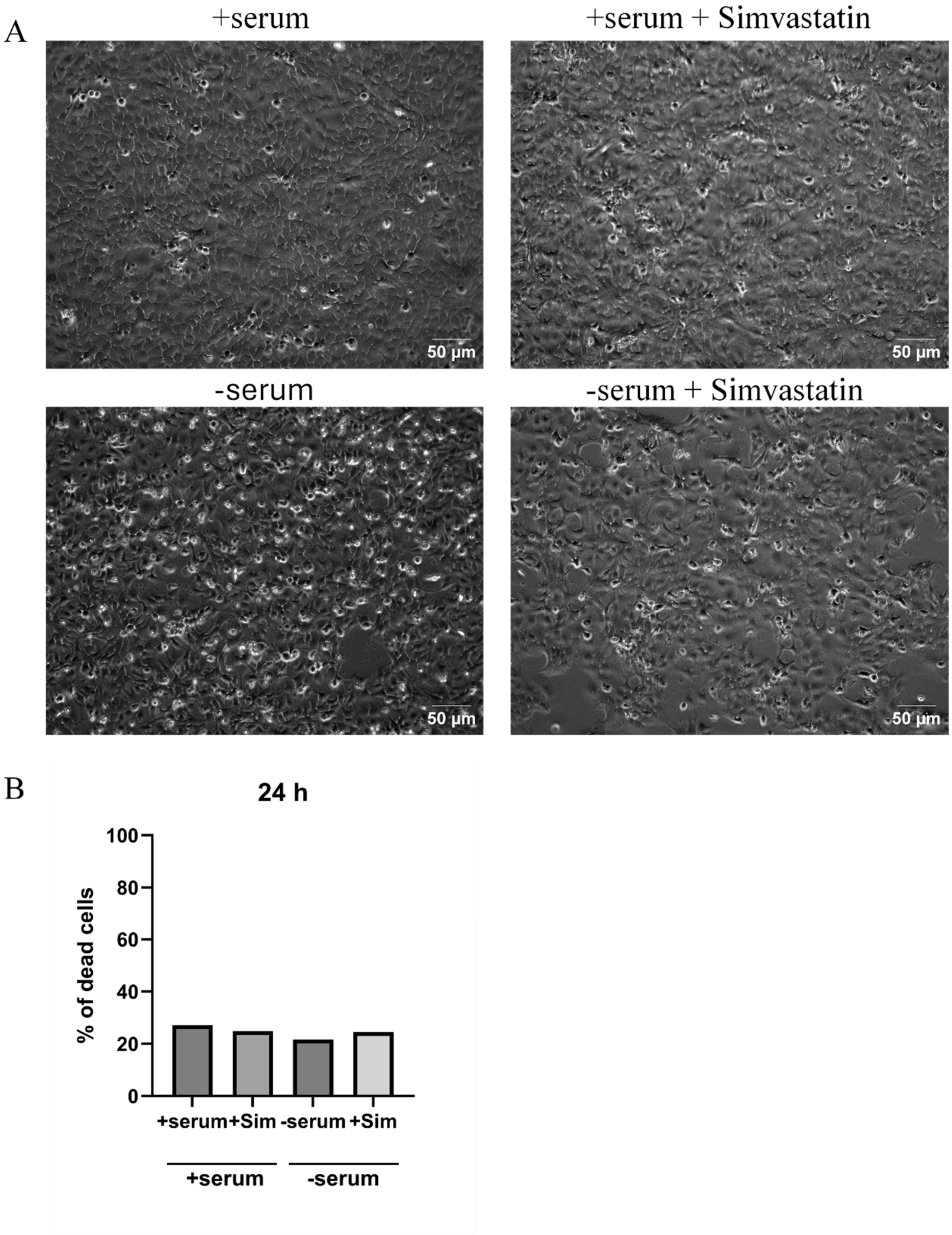

Supplementary Figure 2. Evaluation of the effect of serum deprivation and simvastatin on HaCaT cell viability. (A) Images of HaCaT cells after 24 h of incubation in serum-deprived medium and with simvastatin (20 μM). (B) Flow cytometric analysis of caspase-3/7 activity with 7-AAD revealed that the percentage of dead cells did not change significantly between conditions, indicating that HaCaT cells retained viability even under the combined effects of serum deprivation and simvastatin.

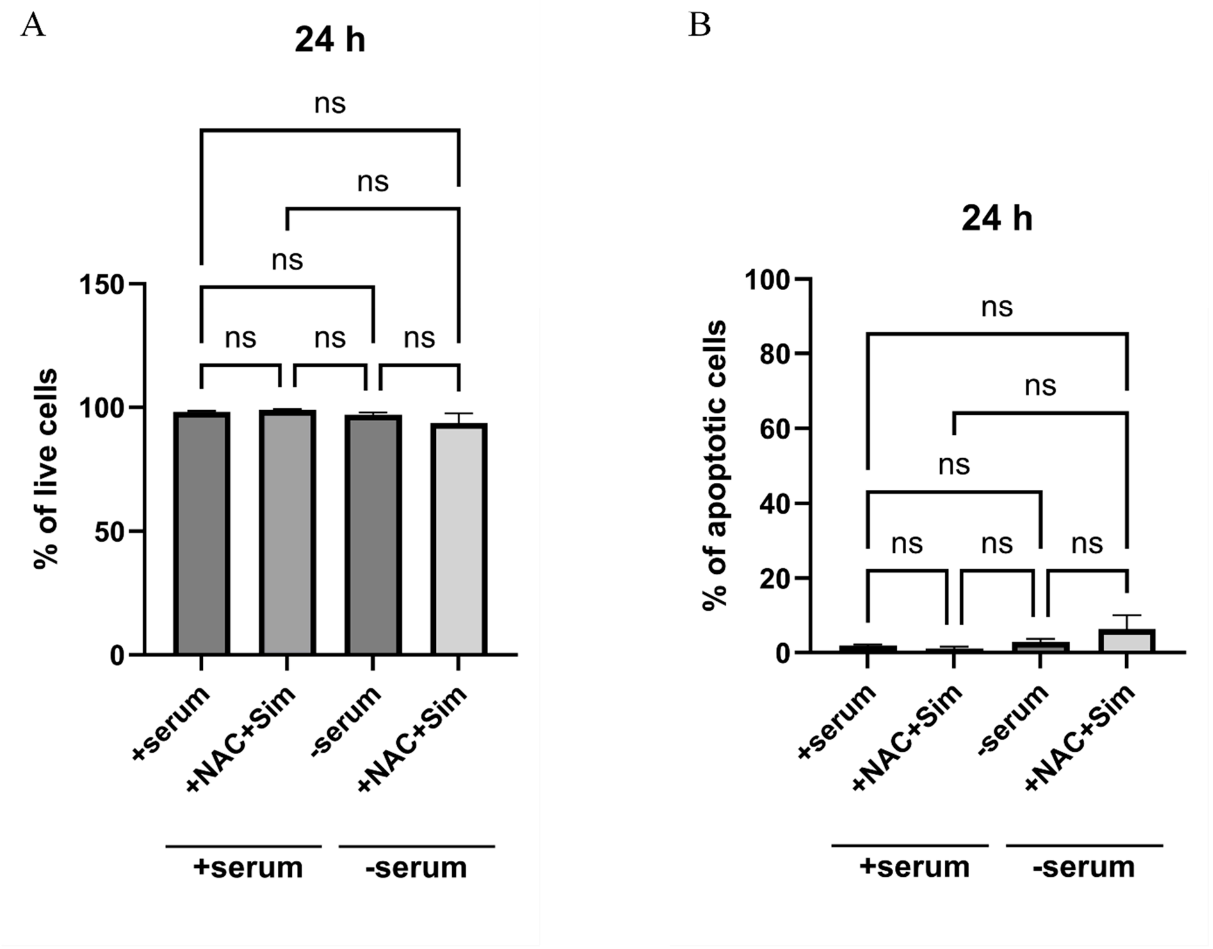

Supplementary Figure 3. Evaluation of the effect of the antioxidant NAC on the viability and apoptosis of H1299 cancer cells treated with simvastatin during serum deprivation. Cells were grown in serum-free medium supplemented with NAC (5 mM) and simvastatin (20 µM) for 24 h. Viability and caspase-3/7 activity with 7-AAD were assessed using flow cytometry. (A) Percentage of viable cells; (B) Percentage of apoptotic cells. Data are presented as mean ± SD from three independent biological experiments (n = 3). Statistical analysis was performed using ordinary one-way ANOVA, which revealed no significant differences between all conditions in viable and apoptotic cell populations (ns). Addition of NAC prevented the simvastatin-induced decrease in viability, indicating the involvement of ROS in the development of apoptosis.

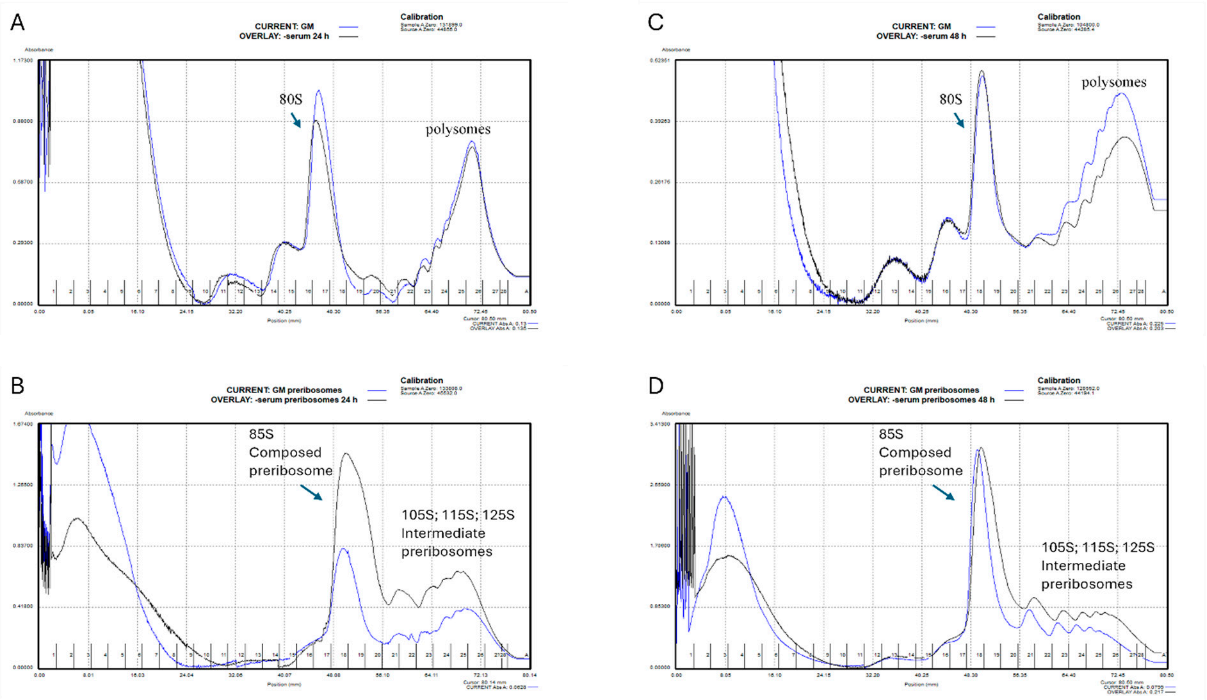

Supplementary Figure 4. Cytoplasmic polysomal (A, C) and preribosomal (B, D) profiles of control (+serum; blue line) and serum-deprived (black line) H1299 cancer cells. H1299 cancer cells were grown in culture medium (with serum, blue line) or in serum-free medium (black line) for 24 h (A, B) and 48 h (C, D). Cells were lysed in magnesium-containing lysis buffer and pre-cleared cytoplasmic fractions, and nuclear fractions were analyzed by sucrose gradient fractionation with detection of nucleic acids of ribonucleoprotein complexes by UV absorption (A254).
